# Chromene flavanones from *Dalea boliviana* as xanthine oxidase inhibitors: *in vitro* biological evaluation and molecular docking studies

**DOI:** 10.3389/fphar.2025.1576390

**Published:** 2025-04-25

**Authors:** Einy Nallybe Bedoya Aguirre, María Daniela Santi, Melisa Fabiana Negro, Javier Echeverría, Margot Paulino Zunini, Mariana Andrea Peralta, María Gabriela Ortega

**Affiliations:** ^1^ Unidad de Investigación y Desarrollo en Tecnología Farmacéutica (UNITEFA-CONICET), Ciudad Universitaria, Córdoba, Argentina; ^2^ Farmacognosia, Departamento de Ciencias Farmacéuticas, Facultad de Ciencias Químicas, Universidad Nacional de Córdoba, Ciudad Universitaria, Córdoba, Argentina; ^3^ Max Planck Institute for Multidisciplinary Sciences, NMR Signal Enhancement group, Goettingen, Germany; ^4^ Departamento de Ciencias del Ambiente, Facultad de Química y Biología, Universidad de Santiago de Chile, Santiago, Chile; ^5^ Área Bioinformática, Departamento de Experimentación y Teoría de la Materia (Detema), Facultad de Química, Universidad de la República, Montevideo, Uruguay

**Keywords:** *Dalea boliviana*, chromene flavanones, xanthine oxidase inhibition, molecular docking, gout, hyperuricemia

## Abstract

**Background:**

Prenylated flavanones represent a structurally diverse class of natural compounds with significant biological potential. Among them, chromene flavanones (CFs) constitute a rare and specialized subgroup with promising therapeutic applications. These molecules have gained attention due to their potential to inhibit xanthine oxidase (XO), a key enzyme involved in oxidative stress-related disorders such as gout and hyperuricemia. Their distinctive structural features, combined with notable bioactivity, make them compelling candidates for further pharmacological exploration. Given their potential relevance, this study focuses on the *in vitro* and *in silico* evaluation of three CFs isolated from *Dalea boliviana* Britton [Fabacea], assessing their capacity to inhibit XO and elucidating key structure–activity relationships (SARs) that contribute to their biological effectiveness.

**Purpose:**

This study aims to investigate the *in vitro* and *in silico* interactions of the chromene flavanones, namely, (*2S*) 5,2′-dihydroxy-6″,6″-dimethylchromeno-(7,8:2″,3″)-flavanone (**1**), (*2S*) 5,2′-dihydroxy-6″,6″-dimethylchromeno-(7,8:2″,3″)-3′-prenylflavanone (**2**), and obovatin (**3**), obtained from *D. boliviana*, with XO, in order to explore their potential as XO inhibitors and their potential therapeutic applications for hyperuricemic diseases.

**Material and Methods:**

XO inhibition by the three chromene flavanones was measured spectroscopically. The relationships between their structures and inhibitory activities were evaluated. Moreover, molecular docking studies were performed to propose the binding modes of the most active natural compounds.

**Results and discussion:**

Compounds **1** and **2** exhibited potent inhibition, with IC_50_ values in the nanomolar range (0.5 ± 0.01 nM and 1.7 ± 0.46 nM, respectively), demonstrating significantly higher activity than allopurinol (AL), the reference inhibitor (IC_50_ = 247 ± 4 nM). In contrast, compound **3** displayed only weak inhibition. SAR analysis revealed that the presence of a chromene moiety in the A-ring, combined with hydroxyl and prenyl groups in the B-ring, played a crucial role in enhancing inhibitory activity. Molecular docking studies confirmed the strong binding affinities of compounds **1** and **2** within the active site of XO (PDB ID: 3NVY), with binding energies of −6.1687 kcal/mol and −6.7820 kcal/mol, respectively. Key stabilizing interactions involved π–π interactions with Phe914 and hydrogen bonding with residues such as Leu873 and Leu1014. These findings highlight the structural features essential for potent XO inhibition and suggest that chromene flavanones represent a valuable scaffold for the development of novel inhibitors. Further molecular dynamics simulations could provide deeper insights into their stability and interaction dynamics, aiding in the rational design of more effective XO inhibitors.

**Conclusion:**

Our findings lead us to propose these chromene flavanones as lead compounds for the design and development of novel XO inhibitors for treating diseases in which exacerbated activity of this enzyme is involved.

## 1 Introduction

Xanthine oxidase (XO, EC 1.17.3.2) is a metalloenzyme responsible for the transformation of hypoxanthine to xanthine and the final generation of uric acid (UA) (Van Hoorn et al., 2002; Chu et al., 2014; Bortolotti et al., 2021). In these pathological processes, hyperuricemia is generated as a relevant risk factor for developing several conditions such as gout, nephrolithiasis, cardiovascular diseases, hypertension, diabetes, and oxidative damage in tissues (Ejaz et al., 2020; Skoczyńska et al., 2020). Although some drugs, such as allopurinol (AL) and oxypurinol—both XO inhibitors—are used for hyperuricemia treatment, they have been associated with some side effects such as hepatitis, nephropathy, and skin rashes (Lin et al., 2015a; Dong et al., 2016; Liu et al., 2021). For these reasons, the investigation of new XO inhibitors is crucial. According to this objective, secondary metabolites such as flavonoids, isolated from plants, showed an important activity as XO inhibitors (Nagao et al., 1999; Van Hoorn et al., 2002; Rasoulzadeh et al., 2009; Lin et al., 2015a; Lin et al., 2015b; Dong et al., 2016; Ejaz et al., 2020; Nguyen et al., 2024). Different studies have reported the relevance of substitution patterns for these compounds to show XO inhibitory activity. Those studies reported the structural–activity relationship (SAR) of different flavonoids, highlighting the influence of the substitution pattern of phenol/hydroxyl groups on this activity (Van Hoorn et al., 2002; Lin et al., 2015a; Dong et al., 2016). Furthermore, our research group demonstrated the impact of methoxyl substituents in the A- and B-rings, present in different natural and semisynthetic flavonoids from *Gardenia oudiepe* Vieill [Rubiaceae] (Santi et al., 2018). Finally, we recently reported other relevant structural requirements related to the presence of a prenyl moiety in flavanones obtained from the *Dalea* genus (Santi et al., 2023).

The genus *Dalea* L. [Fabaceae] is distributed across the Americas, from the southwestern United States to central Argentina and Chile. Among its species, *Dalea boliviana* Britton stands out as, to the best of our knowledge, the only Argentinean species known to contain chromene flavanones (CFs). These specialized metabolites are considered rare due to their limited distribution in the plant kingdom but are notable for their significant biological activities (Yang et al., 2015). Prenylated flavonoids, such as the CFs found in *D. boliviana*, have demonstrated a broad spectrum of biological properties, including antimicrobial effects, inhibition of resistance mechanisms in bacteria and fungi, and tyrosinase enzyme inhibition. These activities underscore their potential as therapeutic agents (Peralta et al., 2019).

Our previous studies highlighted the isolation and characterization of three CFs from *D. boliviana*, which were evaluated for their antifungal activity and their ability to inhibit mushroom tyrosinase (Peralta et al., 2011; Negro et al., 2024). These findings underscore the biological importance of *D. boliviana* as a source of bioactive compounds with potential applications in combating microbial resistance and enzyme inhibition therapies.

Building on the abovementioned findings and taking into account the biological potential of this family of compounds, this work aimed to evaluate the *in vitro* XO inhibitory activity of three CFs from *D. boliviana*, namely, (*2S*) 5,2′-dihydroxy-6″,6″-dimethylchromeno-(7,8:2″,3″)-flavanone (**1**) (Negro et al., 2024), (*2S*) 5,2′-dihydroxy-6″,6″-dimethylchromeno-(7,8:2″,3″)-3′-prenylflavanone (**2**), and the known chromeno (dimethylpyrano) flavanone, obovatin (**3**) (Peralta et al., 2011). In addition, we analyzed *in vitro* and *in silico* structural–activity relationships to determine essential substituents to show XO-inhibitory activity. Finally, molecular modeling methods were carried out to understand the most active compound–enzyme *in silico* binding mode. This investigation shows, for the first time, natural chromene flavanones as lead compounds that could be used for a rational design and development of therapeutic strategies for hyperuricemia-related diseases.

## 2 Materials and methods

### 2.1 Plant material

*Dalea boliviana* Britton [Fabaceae] was collected in February 2019 from Iturbe, located in the Humahuaca Department, Jujuy Province, Argentina (GPS coordinates: 22°58′44″ S, 65°21′13″ W, at an elevation of 3,223 m). A voucher specimen (CORD 1066) was deposited at the Botanical Museum of the National University of Córdoba, Argentina. Prof. Dr. Gloria Barboza from the same institution taxonomically identified the plant material.

### 2.2 Isolation, purification, and identification

Chromene flavanones **1–3** were isolated from the roots of *D. boliviana*. Compounds (*2S*) 5,2′-dihydroxy-6″,6″-dimethylchromeno-(7,8:2″,3″)-flavanone (**1**), (*2S*) 5,2′-dihydroxy-6″,6″-dimethylchromeno-(7,8:2″,3″)-3′-prenylflavanone (**2**), and the known chromeno (dimethylpyrano) flavanone, obovatin (**3**), were purified and identified according to the previously reported methodology (Peralta et al., 2011; Negro et al., 2024). The spectral data of compounds **1–3** are provided in Supplementary Material.

### 2.3 Chemicals

Xanthine (purity: 99.5%), xanthine oxidase (EC 1.14.18.1) from bovine milk (0.04 U/mL), allopurinol (AL; purity: 99%), and HCl (1N) were purchased from Sigma Chemical Co. (St. Louis, MO, United States). All the solvents used were of analytical grade.

### 2.4 *In vitro* xanthine oxidase inhibitory activity

To determine XO inhibition by chromene flavanones, a colorimetric assay was performed, as previously described (Santi et al., 2023). Spectrophotometric measurements were carried out at 295 nm using a Cary Win UV–VIS spectrophotometer (Varian, Inc., Agilent Technologies, Santa Clara, United States), with the formation of UA monitored as an indicator of XO activity. Different concentrations of the CFs (**1**–**3**) or AL (reference inhibitor) were added to the samples before the enzyme addition, and their effect on the generation of UA was used to calculate the percentage of inhibition. A control solution consisting of K_2_HPO_4_/KH_2_PO_4_ buffer was also measured. The inhibition of XO by the compounds was calculated as follows: % inhibition = [(Abs_control_ − Abs_sample_)/Abs_control_] x 100, where Abs_control_ is the absorbance of the control solution and Abs_sample_ is the absorbance of the sample solution.

### 2.5 Calculations and statistics

The XO assays were carried out in triplicate, and the results were expressed as the median ± SEM of three separate experiments. The IC_50_ values were estimated using GraphPad Prism 6.0 software on a compatible computer. The unidirectional analysis of variance (ANOVA), followed by Tukey’s test for multiple comparisons of the results, was performed using GraphPad Prism 6.0 software.

### 2.6 *In silico* structure–activity relationship

The SAR analysis was conducted to qualitatively identify key functional groups that significantly contribute to the inhibitory activity of the chromene flavanones on XO. To perform this analysis, the MOE 2020.22 suite (Chemical Computing Group Inc., http://www.chemcomp.com) (Chemical Computing Group Inc. (2020)) and the MOE SAR tool (Clark and Labute, 2009) were used, which are designed to detect and analyze multiple common scaffolds within small collections of biologically relevant molecules that share similar structural motifs. The input data included molecules with significant XO inhibitory activity. The online report contains potential scaffolds and information on the substitution points on these scaffolds. The algorithm used in this tool was described by Clark and Labute (2009). This approach enabled the identification of critical structural features associated with the observed biological activity.

### 2.7 Molecular docking studies

Molecular modeling studies were conducted to understand the binding mode of new chromene flavanones with XO. The crystal from milk bovine xanthine oxidase (PDB ID: 3NVY), co-crystallized with quercetin at 2.0 Å resolution, was obtained from the Protein Data Bank (http://www.rcsb.org/pdb; Burley et al., 2017). We processed these crystallographic coordinates and performed our docking analyses using the MOE 2020.22 suite (Chemical Computing Group Inc., http://www.chemcomp.com), as previously reported (Santi et al., 2018; Santi et al., 2023; Peralta et al., 2019; Abu-Izneid et al., 2024). In summary, the 3NVY crystal structure was prepared by removing water molecules that were not essential for the interactions of interest. Hydrogen atoms and charges were adjusted using the MMFF94 force field. The 3D structures of chromene flavanones were built and energy-minimized using the AMBER 14 force field. To explore different possible conformations, we generated conformers on the fly during the docking procedure using the LowModeMD conformational search tool (Todorov et al., 2024). Docking experiments considered residues within a 9 Å radius centered on the atoms of quercetin. The Triangle Matcher method was used for initial placement, and the Affinity DG scoring function was applied to evaluate binding affinities (MOE Chemical Computing Group, 2009). We validated our results by reproducing the binding pose of quercetin in the active site (Santi et al., 2023). Graphical representations of the resulting XO–flavonoid complexes were generated using surface maps and ligand interaction tools in MOE (Clark and Labute, 2007).

## 3 Results and discussion

### 3.1 Chromene flavanones as XO inhibitors

**FIGURE 1:**
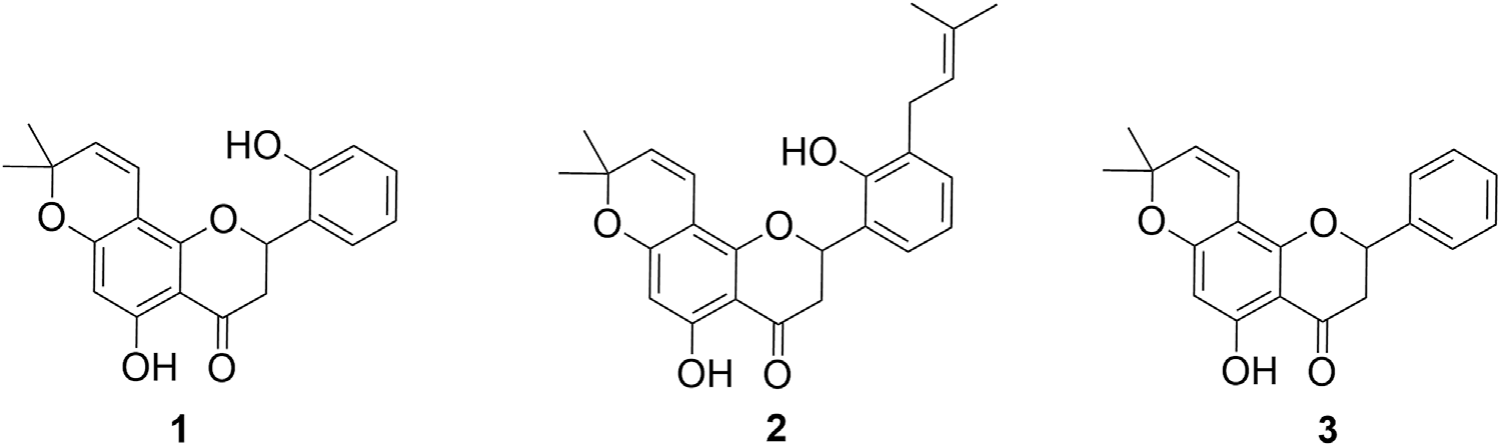
Structures of chromene flavanones isolated from *D. boliviana* (**1**–**3**).

In this study, three CFs, namely, **1**, **2**, and **3** (Figure 1) isolated from *D. boliviana*, were evaluated for their XO inhibitory capacity.

**FIGURE 2:**
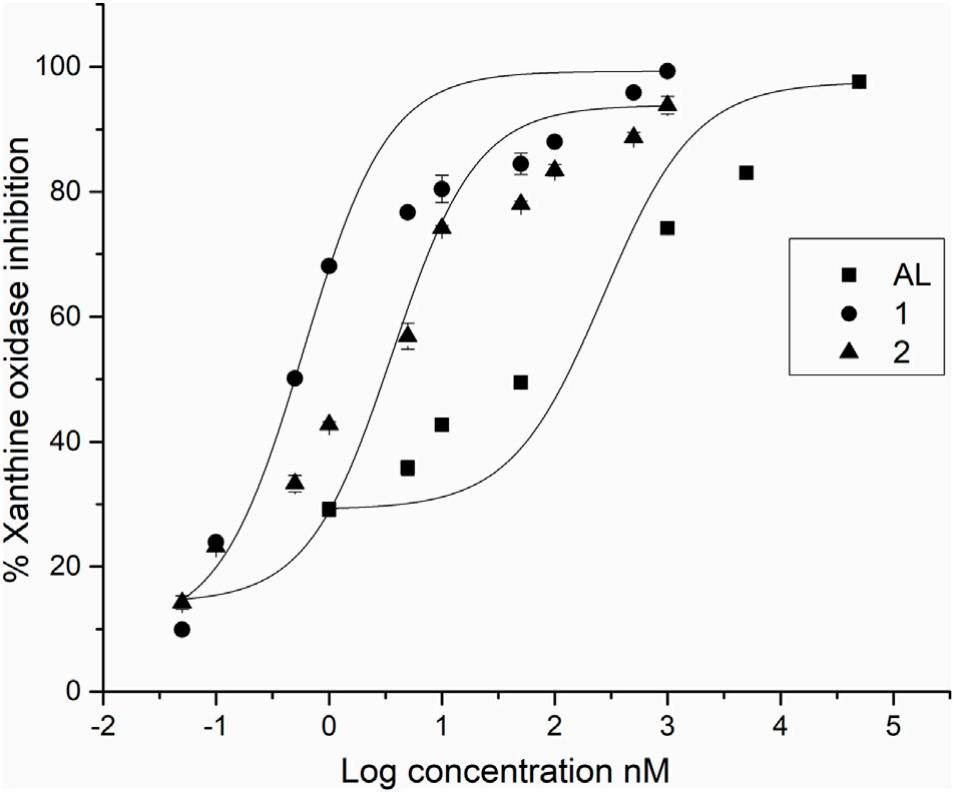
IC_50_ profiles of XO inhibitors.

While all the compounds showed different inhibition potentials, the most active were CFs **1** and **2**. Therefore, their concentration-dependent inhibitory effects, along with that of the reference inhibitor AL, were evaluated using nonlinear fitting of the concentration-response data (Table 1 and Figure 2).

**TABLE 1:** IC_50_ values of CFs and the reference inhibitor (AL) as XO inhibitors.

Compound	IC_50_ (nM) ± SEM
1	0.5 ± 0.01^b^
2	1.7 ± 0.46^b^
3	ND
AL	247 ± 4^a^

ND, not determined (percentage of inhibition: 13.8% at 10 µM).

^a^ Positive control.

^b^ Median ± SEM of at least three determinations. *p* < 0.0001; the values resulted significantly different from the value of allopurinol.

Among the compounds tested, CFs **1** and **2** showed strong XO inhibition activity (with no statistically significant difference between their IC_50_ values), which were approximately 50-fold and 15-fold more potent than AL, the positive control (*p* < 0.001, indicating statistically significant differences in IC_50_ values). On the other hand, CF **3** showed weak inhibition.

### 3.2 Structure–activity relationships analysis

**FIGURE 3:**
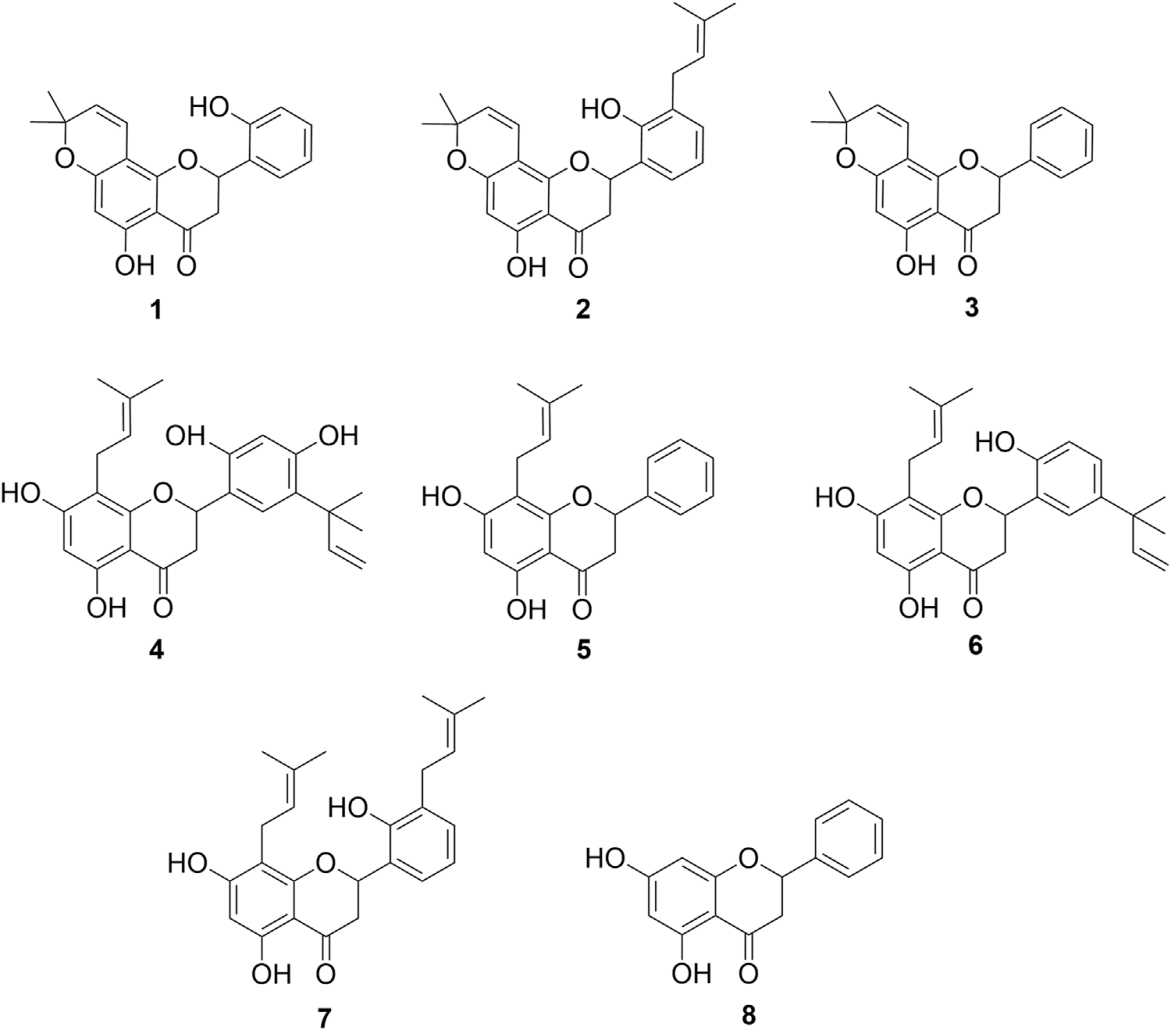
Chemical compounds used in the SAR analysis. **1**: 5,2′-dihydroxy-6″,6″-dimethylchromeno-(7,8:2″,3″)-flavanone, **2**: 5,2′-dihydroxy-6″,6″-dimethylchromeno-(7,8:2″,3″)-3′-prenylflavanone, **3**: obovatin, **4**: 5,7,2′,4′-tetrahydroxy-5’-(1‴,1‴-dimethylallyl)-8-prenylflavanone, **5**: 5,7-dihydroxy-8-prenylflavanone, **6**: 5,7,2′-trihydroxy-5′-(1‴,1‴-dimethylallyl)-8-prenylflavanone, **7**: 5,7,2′-trihydroxy-8,3′-diprenylflavanone, and **8**: pinocembrin.

Although a SAR study requires a large number of structurally related compounds to allow for a relevant and valid structure–activity relationship, the results observed in this work allow us to correlate some structural requirements associated with the activity of the analyzed flavanones. For this reason, *in vitro* and *in silico* SAR studies were conducted to complement and enhance the understanding of these compounds. In the present analysis, the structures reported in this study, along with four prenylated flavanones (**4–7**) obtained from the *Dalea* species and pinocembrin (**8**) (Figure 3), were incorporated along with their IC_50_ data (Santi et al., 2023).

#### 3.2.1 *In vitro* SAR

Some structural requirements for XO inhibition have been identified, such as the presence of hydroxyl groups at positions 5, 7, and 4′ of flavones (Van Hoorn et al., 2002; Lin et al., 2015a; Dong et al., 2016). Our previous research work has contributed additional structural considerations, such as the presence of methoxyl (Santi et al., 2017) and prenyl moieties (Santi et al., 2023) in the A and B rings.

In this study, we extend the data by incorporating chromene flavanones as potential *in vitro* XO inhibitors. Our results indicate that the presence of a chromene moiety in the A-ring enhances XO inhibitory activity compared to the activity showed by prenylated flavanones (in the micromolar range) without this substituent, as previously reported (Santi et al., 2023). Considering the relevant activity observed for compounds **1** and **2**, other specific structural features may also be involved in XO inhibition for these CFs. In this regard, the presence of a 2′ hydroxyl group as the only substituent in the B-ring seems to be crucial for the strong biological capacity of compound **1** as an XO inhibitor. This is supported by a comparison between compounds **1** and **3**, where compound **3** did not show biologically relevant inhibitory activity. According to with the IC_50_ values, the presence of a prenyl moiety in 3′ next to the OH group in 2′ in the B-ring, as observed in compound **2**, also contributes to the enhanced activity, with compounds **1** and **2** showing inhibitory activities in the nanomolar range. Another relevant aspect is the comparison between **2** and 5,7,2′-trihydroxy-8,3′-diprenylflavanone (**7**) (Figure 3), another compound obtained from roots of *D. boliviana*, which was recently evaluated for its XO-inhibitory activity (Santi et al., 2023). These compounds share a core, including a 5-hydroxy in the A-ring and 2′-hydroxy and 3′prenyl moieties in the B-ring. Compound **2** showed a more relevant activity than compound **7**, suggesting that the presence of the chromene moiety in the A-ring of compound **2** contributed to its higher inhibitory activity. Notably, compound **3** did not show any activity despite having a chromene moiety in the A-ring. For these reasons, we consider that the presence of chromene, together with an OH group in the 2′ position and 3-prenyl moiety, led to relevant XO inhibitory activity. Taking all these facts together, we could affirm that the chromene moiety generates a great impact on XO inhibition when specific substitution/s are present in the B-ring. In this type of CFs, the absence of substitution in the B-ring leads to reduced activity.

Considering the information obtained in relation to some structural requirements in order to show the XO-inhibitory activity *in vitro*, we decided to deepen their studies with *in silico* approximations as SAR and molecular docking studies not only to validate our *in vitro* findings but also to determine further details regarding their binding mode.

#### 3.2.2 *In silico* SAR

**FIGURE 4:**
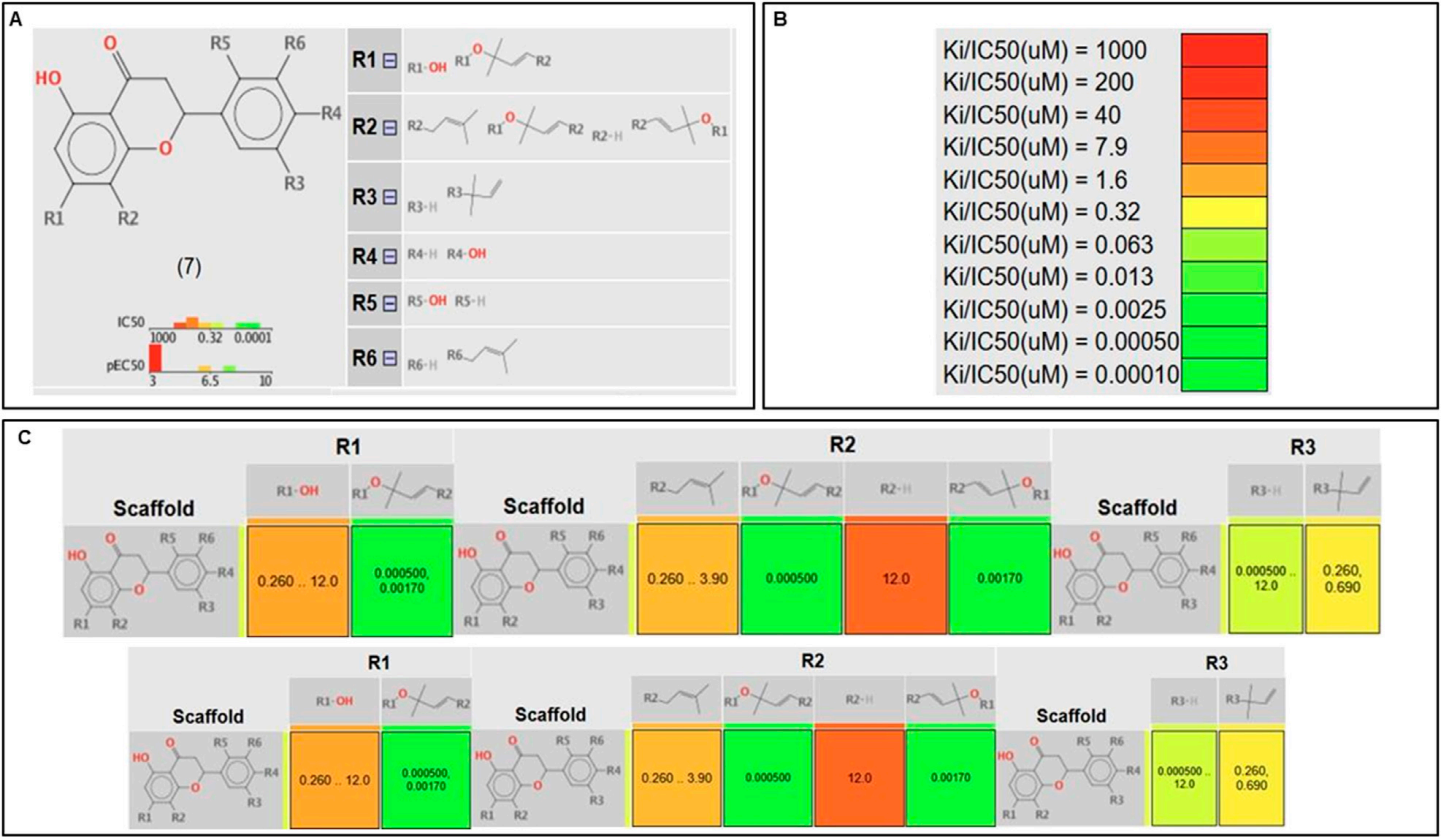
*In silico* SAR. **(A)** Scaffold selected with numbered R-group decorations showing where substituents can be found. **(B)** Heat map color scheme. **(C)** Correlation tables between scaffold and the activity for each R substituent.

The *in silico* SAR tool employed in this study utilizes the core structure of a flavonoid as the scaffold, with substitutions at positions R1–R6 (Figure 4). The analysis evaluates how each substituent affects the biological activity of the compound, measured in terms of its IC_50_ values. The results are visualized through a heat map, where the green color indicates high activity (low IC_50_ values) and the red color represents low activity (high IC_50_ values).

From our analysis, it could be observed that in the A-ring (positions R1 and R2), substitution with a chromene ring leads to highly active molecules. In contrast, bulkier groups, such as alkyl chains or hydroxyl groups, tend to reduce activity, likely due to steric hindrance or flexibility issues that disrupt interactions within the target’s active site. Another relevant finding was the impact of substituents at R5 and R6, corresponding to positions 2′ and 3′ of the B-ring, respectively. The presence of prenyl and hydroxyl groups in these positions enhances activity, potentially through improved solubility or additional hydrogen binding. This observation aligns with findings from Santi et al., who reported that B-ring substituents (e.g., OH groups) establish critical interactions with XO catalytic residues, including Glu802 and Phe914, both of which are crucial for substrate hydroxylation. In our *in vitro* assays, these results were further corroborated, where compounds **1** and **2**, the most active in this study, feature a hydroxyl and prenyl group as substituents in the B-ring.

Finally, at R4 and R3, corresponding to positions 4′ and 5′ on the B-ring, hydroxyls and dimethylallyl groups contribute positively to activity, indicating that not all bulky substituents decrease activity. Overall, the analysis underscores that structural rigidity and substituent size at key positions can foster favorable interactions with the molecular target, emphasizing the importance of rational molecular design in optimizing biological activity.

### 3.3 *In silico* molecular docking studies

**FIGURE 5:**
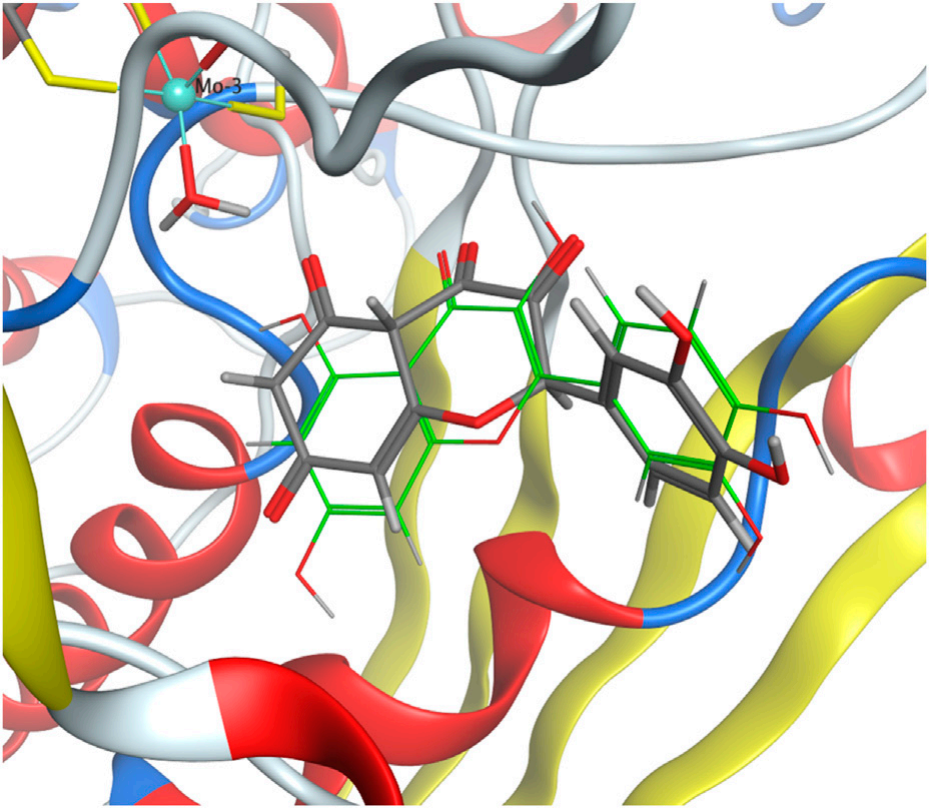
Crystallographic structure of quercetin bound to XO (PDB ID 3NVY) (gray) compared with the docking pose of quercetin from validation studies (green).

The molecular docking protocol was validated by re-docking the native reference ligand, quercetin, into the XO receptor (Figure 5). Five poses were generated to identify the best-evaluated configuration, with a target RMSD value below 2 Å. The final RMSD value of 1.81 Å confirmed that the docking protocol successfully reproduced the ligand’s binding orientation observed in the original crystal structure.

Molecular docking analysis of compounds **1** and **2** was performed using XO (PDB ID: 3NVY) as the receptor. The binding affinity values obtained were −6.1687 kcal/mol for compound **1** and −6.7820 kcal/mol for compound **2**, indicating similar binding energies. Consistently, the experimental IC_50_ values for both compounds showed no significant differences, supporting their comparable biological activities.

The active site of XO comprises key residues, including Glu802, Phe914, Met770, Lys771, and Asn768, which are essential for ligand recognition and stabilization. Leu104, Glu802, Leu 873, and Phe914 play a central role in substrate hydroxylation (Šmelcerović et al., 2017), while Asn768 and Met770 contribute to hydrogen bond formation and proper ligand orientation (Cao et al., 2014).

**FIGURE 6:**
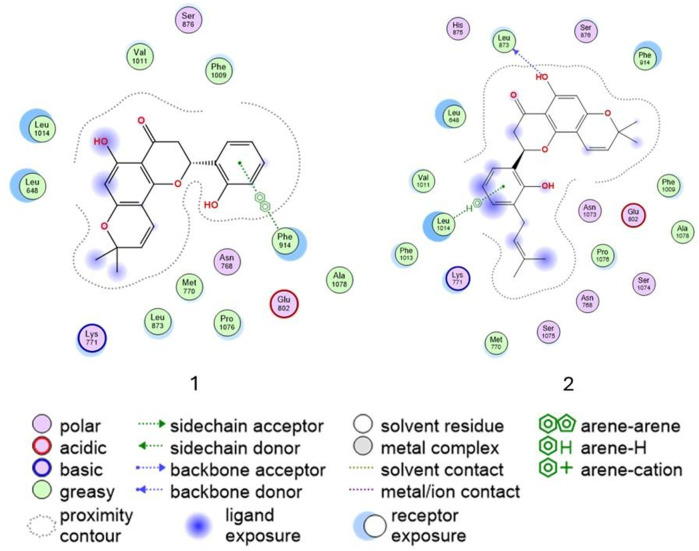
Ligand interaction of compounds **1** and **2** and residues of the active site of xanthine oxidase (3NVY).

The 2D interaction diagrams (Figure 6) illustrate the binding interactions of both ligands within the active site. Compound **1** establishes π–π interactions with Phe914, a residue critical for ligand stabilization, while compound **2** forms π–H interactions with Leu1014 and hydrogen donor interactions between its hydroxyl group on ring A and Leu873. These interactions contribute to proper ligand positioning and binding stability. Overall, the comparable activities of compounds **1** and **2** can be attributed to their ability to form key stabilizing interactions within the active site, particularly involving residues such as Phe914 and Leu1014. Notably, π–π interactions, such as those observed between compound **1** and Phe914, are generally stronger and more stable than π–H interactions. However, both compounds demonstrate favorable binding orientations, explaining their similar biological profiles.

To further investigate these observations, molecular dynamics (MD) simulations could be employed to explore the stability and behavior of the complex of compounds **1** and **2** over time. MD studies would provide insights into the dynamic nature of ligand–receptor interactions, including the durability of key interactions, flexibility within the active site, and potential conformational changes in the enzyme or ligands. As mentioned, MD simulations and advanced energy calculations could enhance our understanding of these systems and guide the rational design of more effective inhibitors.

## 4 Conclusion

In this work, we presented the *in vitro* XO inhibition of three CFs isolated from *D. boliviana*. Compounds **1** and **2** presented a similar activity and were more potent than the reference inhibitor, AL. Compound **3** did not show relevant activity.

This is the first report on the structure–activity relationships of chromene flavanones as XO inhibitors. The relevant XO inhibitory activity of CFs becomes evident compared to that of prenylated flavanones as CFs exhibit activity in the nanomolar range, showing that the presence of the chromene moiety enhances biological activity. Additionally, *in silico* studies such as SAR and molecular docking studies of compounds **1** and **2** showed the relevant interactions between these compounds and relevant amino acid residues of the XO active site. Our findings suggest that CFs 1 and 2 are promising candidates for XO inhibition, either as lead molecules for the rational design of novel XO inhibitors or as part of pharmacological strategies exploring their combined use to assess potential synergistic or additive effects for enhanced biological activity. Further studies are needed to elucidate the pharmacokinetic and pharmacodynamic profiles of compounds **1** and **2**, with the aim of developing new therapeutic agents for disorders associated with excessive XO activity.

## Data Availability

The original contributions presented in the study are included in the article/Supplementary Material; further inquiries can be directed to the corresponding authors.

## Abbreviations

AL, allopurinol; CFs, chromene flavanones; XO, xanthine oxidase.
