# Supplementary material for: Chromene flavanones from Dalea boliviana as xanthine oxidase inhibitors: in vitro biological evaluation and molecular docking studies
Source: Front Pharmacol. 2025 Apr 25;16:1576390. doi: 10.3389/fphar.2025.1576390 (PMC12062022; doi:10.3389/fphar.2025.1576390)
Supplement: Supplementary file 1 [file DataSheet1.docx]

**Chromene Flavanones from *Dalea* *boliviana* as Xanthine Oxidase Inhibitors: *in vitro* biological evaluation and molecular docking studies**

Einy Nallybe Bedoya Aguirre^1,2^, María Daniela Santi^3^, Melisa Fabiana Negro ^1,2^, Javier Echeverría^4*^, Margot Paulino Zunini^5^, Mariana Andrea Peralta^1,2^, María Gabriela Ortega^1,2*^

^1^ Unidad de Investigación y Desarrollo en Tecnología Farmacéutica (UNITEFA-CONICET), Ciudad Universitaria, 5000 Córdoba, Argentina.

^2^ Farmacognosia, Departamento de Ciencias Farmacéuticas, Facultad de Ciencias Químicas, Universidad Nacional de Córdoba, Ciudad Universitaria, Haya de la Torre y Medina Allende, Edificio Ciencias II, Córdoba, Argentina.

^3^ Max Planck Institute for Multidisciplinary Sciences, NMR Signal Enhancement group, Goettingen, Germany.

^4^ Departamento de Ciencias del Ambiente, Facultad de Química y Biología, Universidad de Santiago de Chile, Santiago, Chile.

^5^ Área Bioinformática, Departamento de Experimentación y Teoría de la Materia (DETEMA), Facultad de Química, Universidad de la República, Montevideo, Uruguay.

***Corresponding authors:**

**E-mail address:** Dr. Javier Echeverría (javier.echeverriam@usach.cl); Dra. María Gabriela Ortega ([gortega@fcq.unc.edu.ar](mailto:gortega@fcq.unc.edu.ar))



**Figure 1S.** ^1^H NMR spectrum of compound **1.**



**Figure 2S.**^13^C NMR spectrum of compound **1.**


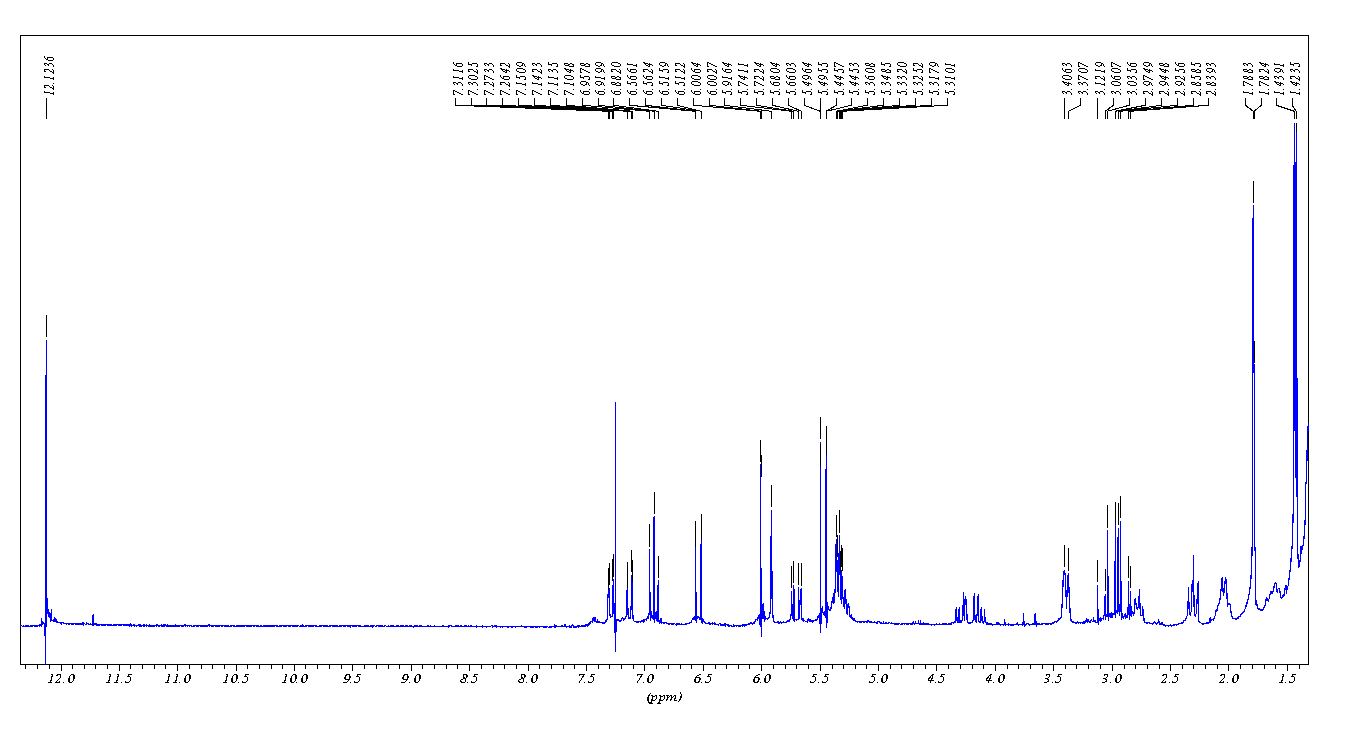


**Figure 3S.** ^1^H NMR spectrum of compound **2.**


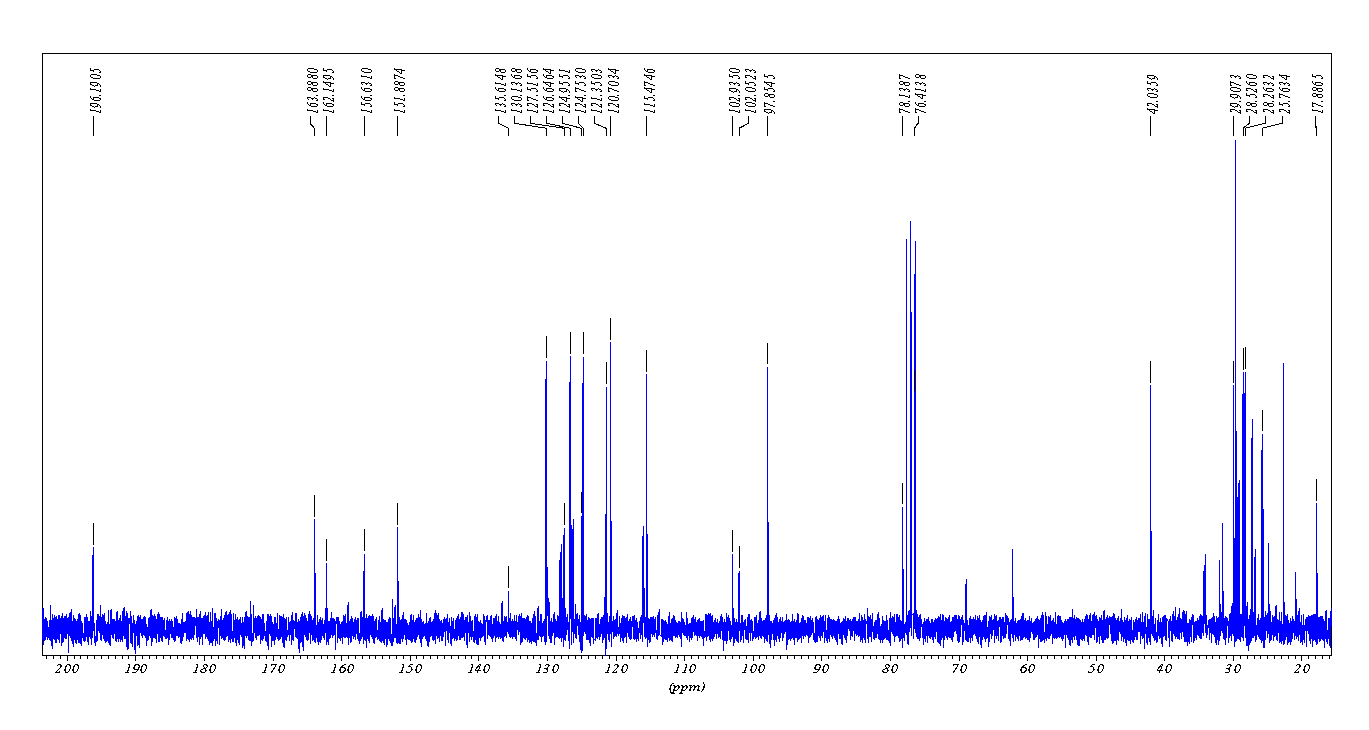


**Figure 4S.**^13^C NMR spectrum of compound **2.**


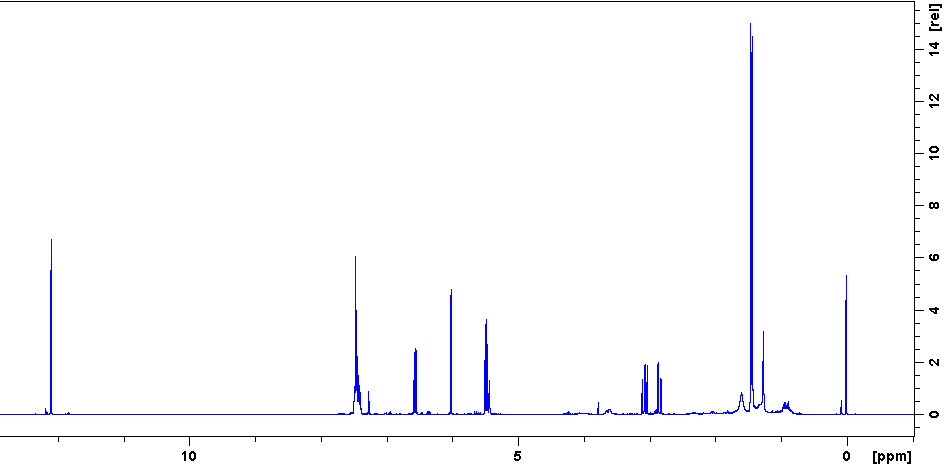


**Figure 5S.** ^1^H NMR spectrum of compound **3.**

**
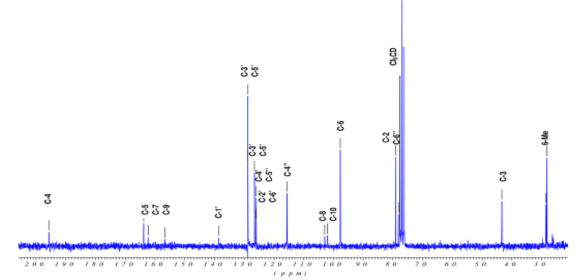
**

**Figure 6S.**^13^C NMR spectrum of compound **3.**
